# Association between 8-OHdG Levels and Eosinophil Counts in Chronic Rhinosinusitis with Nasal Polyps

**DOI:** 10.1055/s-0046-1819639

**Published:** 2026-04-24

**Authors:** Tamara Silva Vieira, Letícia Martins Guimarães, Elisa Carvalho Siqueira, Rafaela Magalhães Braga Amaral, Wilma Terezinha Anselmo-Lima, Edwin Tamashiro, Marina Gonçalves Diniz, Fabiana Cardoso Pereira Valera, Ricardo Santiago Gomez, Carolina Cavalieri Gomes

**Affiliations:** 1 Department of Pathology, Institute of Biological Sciences, Universidade Federal de Minas Gerais (UFMG), Belo Horizonte, MG, Brazil; 2 Department of Ophthalmology, Otorhinolaryngology and Head and Neck Surgery, Faculdade de Medicina de Ribeirão Preto, Universidade de São Paulo (USP), Ribeirão Preto, SP, Brazil; 3 Department of Oral Surgery and Pathology, Universidade Federal de Minas Gerais (UFMG), Belo Horizonte, MG, Brazil

**Keywords:** chronic rhinosinusitis with nasal polyps, HRAS, NRAS, oxidative stress, eosinophil, 8-OHdG

## Abstract

**Introduction:**

Oxidative stress plays an important role in the pathogenesis of chronic rhinosinusitis with nasal polyps (CRSwNP). The 8-Hydroxydeoxyguanosine (8-OHdG) is a major oxidative DNA adduct, and its levels have been noted as a triggering factor for inflammation-related carcinogenesis, but have not been assessed in CRSwNP. The MAPK/ERK pathway importance in the pathogenesis of CRSwNP has previously been reported. Mutations in *RAS* family genes occur in neoplastic and non-neoplastic proliferative lesions, and it has been previously demonstrated that *KRAS* mutations do not occur in CRSwNP.

**Objective:**

To assess the levels of 8-OHdG in nasal polyps occurring in CRSwNP, to associate these levels with clinicopathological parameters, and to investigate *HRAS* and *NRAS* hotspot mutations in the same samples.

**Methods:**

Both the *HRAS* (codons 49 and 61) and *NRAS* (codon 61) mutations were investigated through Sanger sequencing, and the levels of 8-OHdG were determined using ELISA in 14 freshly collected snap-frozen samples of nasal polyps occurring in CRSwNP. The associations between the levels of 8-OHdG and patients' age, presence of asthma, and eosinophil counts in nasal polyps were tested.

**Results:**

No mutations were detected. The association between higher levels of 8-OHdG and higher eosinophil counts was observed, whereas there was no association between it and age or the presence of asthma.

**Conclusion:**

Based on the results in the present cohort, higher levels of 8-OHdG, indicative of oxidative stress, are associated with higher eosinophil counts in CRSwNP. Additionally, *HRAS* and *NRAS* mutations do not occur in nasal polyps or occur at a very low frequency.

## Introduction

Chronic rhinosinusitis is a common condition in adults in most parts of the world, occurring in approximately 12% of the population. 1 2 3 Chronic rhinosinusitis is broadly classified into two phenotypes: with or without nasal polyps. 4 Chronic rhinosinusitis with nasal polyps (CRSwNP) is usually characterized by the presence of bilateral polyps that can be visualized endoscopically in the middle meatus. 4 5 6 Nasal polyps are frequently associated with asthma. The coexistence of these two comorbidities indicates worse prognosis. 7

The pathogenesis of CRSwNP has been extensively investigated, but it remains elusive whether nasal polyps harbor genetic alterations. 4 5 6 Also, it involves the participation of multiple inflammatory cells and mediators, with a skewed response toward type 2 inflammatory pattern in the stroma, including IL-4, IL-5, IL-13, and eosinophilic infiltration, 8 that may be precipitated by epithelium barrier dysfunction. 9

The imbalance between the production and degradation of reactive oxygen species (ROS) causes oxidative stress, which, in turn, leads to nasal epithelium barrier impairment. 10 Oxidative stress plays a role in the pathogenesis of nasal polyps. 11 12 13 For instance, oxidative DNA damage may modify bases with the risk of inducing mutations. 11 The 8-Hydroxydeoxyguanosine (8-OHdG) is a major oxidative DNA adduct, and it has a central role in senescence, carcinogenesis and other disease processes. Although increased 8-OHdG level has been pointed out as a triggering factor for inflammation-related carcinogenesis, 14 it has not been evaluated in CRSwNP yet.

Inflammation in CRSwNP affects MAPK/ERK signaling pathway, 15 which regulates various cellular biological behaviors. 16 The *KRAS* , *BRAF* , and *EGFR* genes are part of the MAPK/ERK pathway and can be mutated in some benign and/or inflammatory lesions, 17 though such cases do not occur in nasal polyps. 18 It is plausible to speculate that other MAPK/ERK pathway genes may play a role in the pathogenesis of nasal polyps in patients with CRSwNP, since *RAS* gene mutations have been reported in head and neck squamous cells carcinomas and, most importantly, in nasal tract lesions, such as oncocytic sinonasal papilloma and sinonasal squamous cell carcinoma associated with oncocytic sinonasal papilloma. 19 20 Of note, *HRAS* mutations have been reported in cutaneous pyogenic granulomas, 21 which are non-neoplastic proliferative lesions.

Based on the aforementioned information, the present study aimed to assess 8-OHdG levels in nasal polyps from patients with CRSwNP and to associate the results with patients' age, presence of asthma and the number of eosinophils in nasal polyps. Additionally, we investigated *HRAS* and *NRAS* hotspot mutations in nasal polyps from patients with CRSwNP.

## Methods

### Samples

A convenience sample of fourteen freshly collected and frozen nasal polyp samples from patients with CRSwNP was included in this study. We defined CRSwNP according to the diagnostic criteria established by the European Position Paper on Rhinosinusitis and Nasal Polyps 2020. 5 The study was approved by the Ethics Research Committee of the authors' University (protocol: 58148722.5.1001.5149). The collected samples were frozen at −70°C until the moment of DNA extraction. This cohort of samples was used in a previous study. 18

### DNA Extraction and Sanger Sequencing

Genomic DNA (gDNA) extraction from nasal polyps was performed using the DNeasy blood and tissue kit (Qiagen Inc.) according to the manufacturer's instructions. The quantity and purity of DNA were assessed with a spectrophotometer (NanoDrop 2000; Thermo Fisher Scientific).

### Polymerase Chain Reaction (PCR) and Sanger Sequencing

The PCR was performed for the amplification of the hotspot mutation sites at *HRAS* (codons 49 and 61) and *NRAS* (codon 61), using a MyTaq HS Red Mix, 2x (Bioline Reagents Ltd.), and running it on the Mastercycler PRO (Eppendorf SE). The PCR products were submitted to electrophoresis in a 1.5% agarose gel labeled by fluorescence (SYBR Safe DNA gel stain; Invitrogen, Thermo Fisher Scientific) and visualized in a transilluminator under UV light, purified using isopropanol and Sanger sequenced using the Big Dye Terminator v3.1 Cycle Sequencing Kit (Applied Biosystems). Capillary electrophoresis was performed in the ABI 3130 DNA Analyzer (Applied Biosystems).

All PCR products were sequenced bidirectionally. The chromatograms were visualized and manually inspected through the SnapGene software (GSL Biotech), using the reference sequences *HRAS* : NM_005343 and *NRAS* : NM_002524. The full sequences of each amplicon were manually inspected in search of other possible mutations, not only the hotspot ones.

### Measurement of 8-OHdG Levels

The 8-OHdG is a prominent DNA oxidative modification that can arise from the hydroxylation of deoxyguanosine residues. The levels of 8-OHdG were determined using an enzyme-linked immunosorbent assay (ELISA). For its detection, 1000ng of gDNA of each nasal polyp sample were used with the OxiSelect Oxidative DNA Damage ELISA Kit (8-OHdG Quantitation, Cell Biolabs), following the manufacturer's instruction and all samples were run in duplicate. Absorbance was measured at 450nm using an Epoch Microplate Spectrophotometer (BioTek Instrumentals Inc.), and the concentrations of 8-OHdG in the samples were determined by comparing them to a pre-established standard curve.

### Statistical Analyses

Statistical analysis for the 8-OHdG results was performed using the software GraphPad Prism for Windows (GraphPad Software Inc.), version 10.1.0. Data normality was evaluated using Shapiro–Wilk test. The following clinical variables were compared between the groups: patients' age (younger versus older than the median age), count of eosinophils per high-power field (HPF: lower versus higher than the median) and associated asthma (yes versus no). A cut-off value of 65 eosinophils/HPF was established using the median count across all samples, allowing dichotomization for subsequent analyses.

According to the criteria established by the 2020 European Position Paper on Rhinosinusitis and Nasal Polyps, 5 10 eosinophils per HPF (400x) or higher determine the eosinophilic clinical phenotype. The comparisons were performed using independent samples *t* -test or Mann-Whitney test, according to data distribution. Pearson's test was used for the correlation between 8-OHdG levels and eosinophil counts. Values of *p <* 0.05 were considered statistically significant.

## Results

### Sample Characterization

The median age of patients was of 45.5 (range: 24–65) years. There were five patients with associated asthma. The median eosinophils number per HPF among the samples was 65, and 11 cases were classified as eosinophilic phenotype. 5

Furthermore, 13 participants underwent previous corticosteroid treatment, while information regarding this aspect was unavailable for one individual. Table 1 shows the clinical features and eosinophil counts per sample.

**Table 1:** Clinical features from patients with CRSwNP and eosinophils counts per nasal polyp sample

Sample #	Age (years)	Eosinophils (number per HPF)	Asthma
1	45	100	Yes
2	65	5	Yes
3	44	80	No
4	32	162	No
5	26	120	No
6	58	50	Yes
7	36	50	Yes
8	54	100	No
9	44	80	No
10	46	15	Yes
11	47	100	No
12	56	20	No
13	24	3	No
14	60	0	No

**Abbreviations:** CRSwNP, chronic rhinosinusitis with nasal polyps; HPF, high-power field.

### Sanger Sequencing

**Fig. 1:**
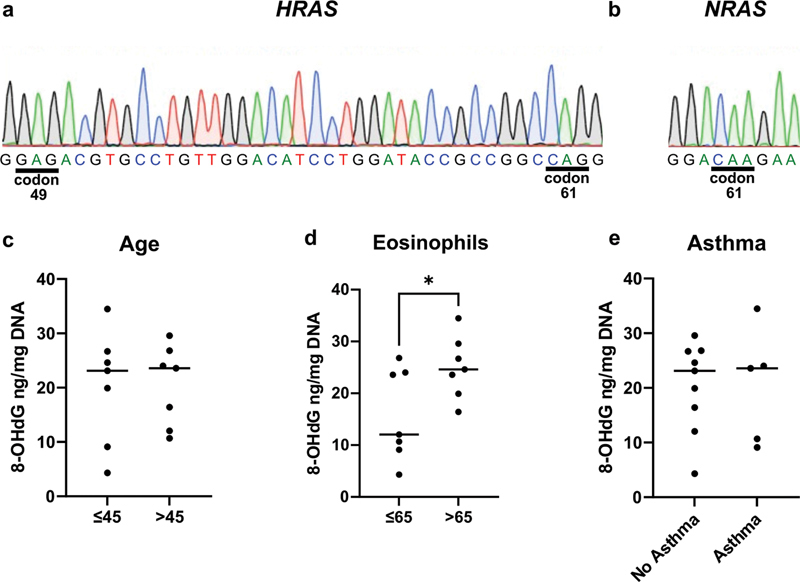
Representative chromatograms of the gene regions evaluated in the nasal polyps and comparison of 8-OHdG levels in samples according to clinical parameters and eosinophil counts. ( **A** ) Screenshots from sequencing chromatograms showing wild-type sequences for the analyzed regions of *HRAS* (codon 49 and 61) and ( **B** ) *NRAS* (codon 61). ( **C** ) Comparison between the groups according to patients' age (younger vs older than the median). ( **D** ) Comparison between the groups according to eosinophil counts (higher vs. lower than the median). Samples with >65 eosinophils per HPF presented a higher concentration of 8-OHdG than the group with ≤65 ( *p* < 0.05). ( **E** ) Comparison of 8-OHdG levels between samples from patients with and without asthma. *When *p* < 0.05, the difference was statistically significant.

All samples showed wild-type sequences for *HRAS* (codons 49 and 61; Fig. 1A ) and *NRAS* (codon 61; Fig. 1B ). Importantly, manual inspection revealed the intronic *NRAS* SNP rs969273 in 13 samples. This SNP clinical significance is benign.

### Measurement of 8-OHdG

There was no significant difference in the 8-OHdG levels between older and younger patients (≤ 45 versus > 45 years; Fig. 1C ). Furthermore, there was no difference in levels between the eosinophilic and noneosinophilic polyps ( *p* = 0.3571; median: 23.59 versus 10.67 ng/mg DNA), but the group of samples with > 65 eosinophils per HPF showed an increased concentration of 8-OHdG when compared with the group ≤65 ( *p* < 0.05; median: 12.06 versus 24.6 ng/mg DNA), as shown in Fig. 1D .

There was positive correlation between the levels of 8-OHdG and the number of eosinophils per HPF (R = 0.5316), however the correlation was not statistically significant ( *p* = 0.050). The levels were similar between samples from patients with associated asthma and those without it ( Fig. 1E ).

## Discussion

Previous in vitro studies showed that pharmacological treatment decreases nasal polyp cell proliferation and increases apoptosis by inhibiting MAPK/ERK signaling pathway. 15 On top of that, another study showed the importance of MAPK/ERK activation in ciliogenesis and cilia function in CRSwNP, further confirming its importance. 22

In the context of MAPK/ERK signaling pathway alterations, mutations in the *RAS* genes ( *KRAS* , *HRAS* and *NRAS)* have been associated with hyperproliferative disorders and cancer, and in tumors they most often occur in hotspot codons. 23 24 For instance, mutations in *KRAS* have been reported in sinonasal neoplasms. 19 Although one study suggested that there are *KRAS* mutations in nasal polyps, 25 we could not detect any in the polyps associated with CRSwNP. 18

Considering that nasal polyps in CRSwNP occur in the context of inflammation, which is known to predispose DNA to mutation, and taking into account that MAPK/ERK pathway importance in the pathogenesis of CRSwNP has previously been reported, 15 22 we assessed *HRAS* and *NRAS* hotspot mutations in nasal polyps. There have been reports of *NRAS* mutations in lymphoproliferative autoimmune disease, highlighting its importance in the inflammatory context. 26 Notably, the predominant *RAS* isoform mutated in squamous cell carcinomas of the head and neck is *HRAS* , with a frequency ranging from 4 to 8%. 27 Additionally, in Costello syndrome, in which *HRAS* mutations are the underlying genetic cause, cutaneous papilloma occurs mainly in the perinasal regions, further reinforcing a role for these mutations in the development of benign proliferative lesions. 28 A previous study reported *HRAS* codon 12 mutation in 1 out of 23 nasal polyps. 25 However, in the present study, all samples showed wild-type sequences for both, *HRAS* and *NRAS* mutations. Considering the above discussed, it seems that mutations in *RAS* genes are not part of the pathogenesis of nasal polyps in CRSwNP.

Oxidative stress can lead to mutation and DNA damage, which can be a predisposing factor for cancer and age-related disorders. 29 Environmental exposure and lifestyle factors, such as smoking, are key sources of oxidative stress. 30 There is a strong relationship between oxidative stress and the pathogenesis of nasal polyps 11 31 and, overall, its severity is significantly correlated with the severity of the symptoms related to CRSwNP. 32 We assessed the levels of 8-OHdG in nasal polyps, since this is an important oxidative DNA adduct, and tested their association with clinical parameters (patients' age and associated asthma), as well as with the eosinophil counts. The age of the patients did not show association. On the other hand, elevated levels of oxidative stress markers are observed in individuals with asthma, and these markers are correlated with the severity and phenotype of the disease. 33 34 Nevertheless, in our study, polyps from CRSwNP showed similar levels of 8-OHdG irrespective of the presence of asthma.

Since eosinophils are a pathological landmark of CRSwNP, 35 36 37 in the present study we evaluated the association between the number of eosinophils and 8-OHdG levels, and the data analysis showed a relevant association. There is extensive epithelial cell disruption in CRSwNP, 9 38 associated with oxidative stress (8-OHdG), probably leading to the overexpression of TH2 cytokines (IL-4, IL-5, and IL-13) and activation and recruitment of eosinophils. 8 39 40 41 Interestingly, the eosinophil cationic protein originates from the activated eosinophils, and a previous study showed that its values have been shown to correlate with nasal obstruction, congestion, and rhinorrhea, which indicate the severity of nasal polyposis. 32 As we observed an association between eosinophil counts and 8-OHdG levels, future studies should explore whether this oxidative stress marker is related to the clinical manifestations, which was beyond the scope of the present work.

The retrospective nature of the current study poses limitations to the interpretation of the results. For instance, patients underwent corticosteroid therapy before surgery, which might have led to an underestimation of the degree of eosinophilic inflammation. Also, it is difficult to obtain precise information regarding corticosteroid regimen (dose, duration, and timing).

## Conclusion

The present study assessed the association between 8-OHdG levels and clinical features, and eosinophil counts in nasal polyps in CRSwNP. We also interrogated *NRAS* and *HRAS* hotspot mutations in these samples. On the basis of our results, neither mutation occurs in nasal polyps, or if they do, they occur at a very low frequency. Importantly, in the present cohort we observed association between higher levels of 8-OHdG and higher eosinophil counts. This finding might stem from oxidative stress induced epithelial dysfunction, triggering TH2 cytokine production and eosinophils recruitment.
